# Exploring the Value of Real-Time Medication Adherence Monitoring: A Qualitative Study

**DOI:** 10.3390/pharmacy11010018

**Published:** 2023-01-18

**Authors:** Sadaf Faisal, Jessica Ivo, Sarah Abu Fadaleh, Tejal Patel

**Affiliations:** 1 School of Pharmacy, University of Waterloo, Kitchener, ON N2G 1C5, Canada; 2 Centre for Family Medicine Family Health Team, Kitchener, ON N2G 1C5, Canada; 3 Schlegel-University of Waterloo Research Institute of Aging (Schlegel-UW RIA), Waterloo, ON N2G 0E2, Canada

**Keywords:** medication adherence, smart technology, medication adherence monitoring, dispensing technology, value

## Abstract

Smart adherence products enable the monitoring of medication intake in real-time. However, the value of real-time medication intake monitoring to different stakeholders such as patients, their caregivers, clinicians, and insurers is not elucidated. The aim of this study was to explore the value different stakeholders place on the availability of smart adherence products and access to real-time medication intake data. A qualitative study design using semi-structured one-on-one virtual interviews was utilized. Schwartz’s theory of values provided the foundation for the interview questions, data were analyzed using Braun and Clark’s thematic analysis framework, and findings were mapped back to the constructs of Schwartz’s theory of values. A total of 31 interviews with patients, caregivers, healthcare providers, and representatives of private or public insurance providers were conducted. Three themes and ten subthemes were identified. Themes included perceptions of integrating smart medication adherence technologies and real-time monitoring, technology adoption factors and data management. Stakeholders place different values based on the motivators and goals that can drive product use for daily medication management. Stakeholders valued the availability of real-time medication taking data that allow clinicians to make timely data-driven recommendations to their patients that may improve medication management for patients and reduce the caregiver burden.

## 1. Background

Medication adherence is a significant healthcare challenge worldwide. Studies have shown that, in developed countries, more than half of patients with chronic illnesses do not take their medications as recommended by their healthcare provider [1,2]. Poor medication adherence can result in the non-optimal treatment of chronic disease, leading to increased emergency room visits, frequent re-hospitalization, poor disease outcomes, and a significant financial burden on healthcare systems [3,4].

The accurate and timely measurement of medication taking is imperative for clinicians to identify non-adherence and make pharmacotherapy decisions [5]. However, no reliable measure of medication intake and adherence exists. Currently, frequently used adherence measurements include counting pills, assessing pharmacy refill data, or patient self-reporting through interviews or questionnaires [6,7]. However, these are surrogate markers of overall adherence and do not provide data related to the time of administration, dose taken or persistence. In the last two decades, there has been an increased influx of technology in the healthcare field, including smart products with real-time medication intake monitoring. These products are being developed to address non-adherence, support a patient’s medication-taking behavior, and track real-time medication intake data [8,9,10,11]. Smart products collect medication intake data remotely through various means of connectivity including Bluetooth, Long-Term Evolution (LTE), Wireless Fidelity (Wi-Fi), Radio Frequency Identification (RFID) or Near Field Communication (NFC), which allows the data to be accessible to the patient, caregivers or healthcare providers in real-time [8,9,12,13]. When a patient interacts with the product by either opening the medication vial or breaking the blister package, the medication access event is captured by the product with a time and date stamp [9]. An example of such smart adherence products is the Wisepill RT2000 dispenser, available commercially amongst many others [13]. Though these products do not confirm medication ingestion, they provide more information about a patient’s medication-taking behavior in real-time, which allows for the early identification of medication non-adherence [8,9].

The data collected through these products may not only be used to address non-adherence in a timely manner and identify a patient’s medication-taking behavior, but may also help clinicians make pharmacotherapy decisions, such as dose optimization or therapy modifications. For example, suppose the real-time data collected through an adherence product show that a patient is consistently not taking their medications at a specific time of the day. In that case, these data can be used during clinical encounters with patients to discuss their adherence patterns, explore the reasons for non-adherence at that time, and develop personalized strategies to improve medication intake instead of increasing the dose or changing the therapy. Additionally, most of these products also incorporate alarms and messaging systems to remind patients to take their medications when their dose is due [8,12,13]. Other features that may be offered by such products include their capacity to organize complex regimens, connect with clinicians or caregivers to monitor medication intake, automatically dispense medications, and restrict access for the safe use of medications [13]. These additional features may help patients incorporate behavior changes related to their medication administration.

A literature review conducted in 2019 identified more than 50 smart products that can record a patient’s medication intake in real time [13]. Another scoping review identified that these products had marked differences in the measurement and reporting of adherence [14]. A recent narrative review reported 79 medication adherence technologies, including electronic pill boxes, pill bottles, blister packages, and many other products that can record medication taking in real time [9]. In 2019, Steinkamp et al. conducted a systematic scoping review exploring technology-based medication adherence interventions used in mental health and substance abuse disorder and identified 34 studies that utilized a smart pill container for the accurate measurement of adherence [15].

A growing body of research is exploring the usability, user experience, and workload involved in using these products by various patient populations, including older adults and people with chronic diseases [16,17,18,19]. Studies have also explored the views and preferences of healthcare providers about these products and factors affecting the offering of such products for patients [20,21,22]. Although these studies reported participants’ perspectives on smart medication adherence products, none of them explored participants’ values related to the availability of real-time monitoring data in a detailed manner.

These real-time medication adherence technologies are available to various stakeholders including patients, caregivers, and healthcare providers such as pharmacists and physicians. If a patient chooses to adopt one of these medication adherence technologies for their daily medication management, one or more of these stakeholders will likely be involved in some capacity. Similarly, insurance providers or payors are another stakeholder group with an interest in the adoption of these technologies as they may be called upon to provide insurance to cover the cost of acquiring and using such products on a long-term basis. At present, limited products are being reimbursed by private or public payors in Canada, and as such it is essential to understand and explore the views and values insurance providers or payors have regarding these products. Therefore, it is integral to understand what these different stakeholders value about the availability of real-time medication intake data provided through smart adherence products. Hence, we designed a qualitative study using Schwartz’s theory of values framework to explore stakeholders’ values about smart adherence products and the availability of real-time medication intake data.

## 2. Objectives

The objective of the study was to explore what stakeholders value about smart adherence products and the access to real-time medication intake data.

Stakeholders: For this study, stakeholders were defined as patients, caregivers, community pharmacists, pharmacy owners, physicians, and private or public insurance providers.

Value: Given the lack of consensus for the definition of “value”, for this study, we defined value as “the worth, usefulness, or importance of someone or something [23]”.

## 3. Methods

### 3.1. Study Design

This study used a qualitative study design with semi-structured one-on-one interviews conducted virtually due to COVID pandemic-related restrictions. The Consolidated Criteria for Reporting Qualitative Research Studies Checklist (COREQ) was used to report the study, see Figure S1 [24].

### 3.2. Ethics Approval

The study received ethics approval from the University of Waterloo Clinical Research Ethics Board (ORE # 43387), Canada. All participants provided consent prior to the interviews. The study was conducted in Canada between August 2021 and January 2022.

### 3.3. Theoretical Framework

**Figure 1:**
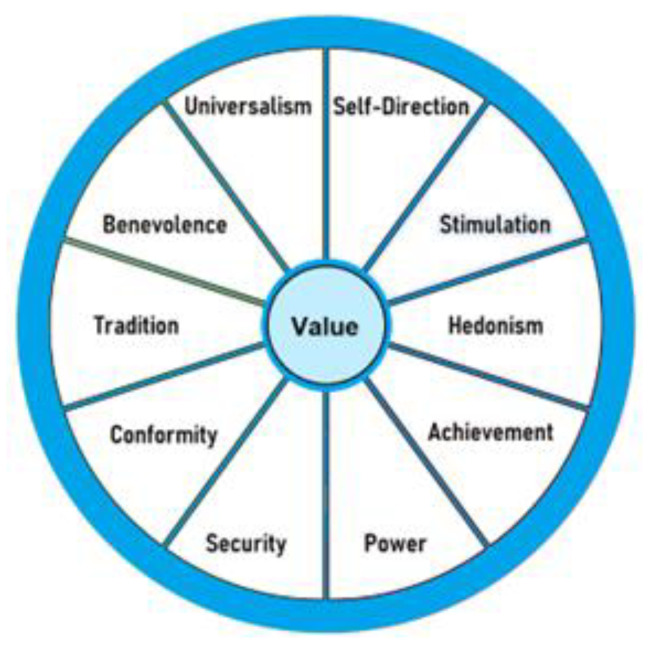
Values of the Schwartz’s theory of values framework [29].

The Schwartz’s theory of values framework was utilized to inform the development of the interview guide and qualitative analysis. Schwartz’s theory of values is a social-psychological theory that identifies ten broad personal values, which are differentiated by underlying goals or motivation (see Figure 1) [25,26]. The term ‘*values*’ represents “meaningful beliefs, principles, or standards of behavior, referring to desirable goals that motivate action [25]”. The framework of values described by Schwartz has been used to determine the impact of “personal values” on various health-related behaviors in previous studies [27,28]. The interview guide was developed to explore these ten personal values in the context of smart adherence products and real-time medication intake data availability. See Table S1 for the questions included in the interview guide.

### 3.4. Recruitment and Sampling

A purposive sampling strategy was used to recruit participants. Sample sizes are not normally calculated for qualitative studies and tend to be smaller in order to allow for the in-depth analysis of individual cases, which is a central aspect of this research approach. Whether one has reached an adequate sample size is usually determined by data saturation, i.e., the point at which qualitative data analysis does not elucidate any new information. We aimed to recruit a sample size of 30 participants (5 from each stakeholder group) initially and planned to continue recruiting until we reached data saturation. The study was advertised through social media (the University of Waterloo School of Pharmacy’s website, Facebook, and Twitter page), researchers’ professional networks, and by contacting previous study participants who had shown interest in participating in future research projects. Participants were eligible to participate in the study if they were: (1) a patient taking at least one medication per day, (2) an informal caregiver such as a family member, friend, or neighbor who was directly involved in assisting a patient with medication management, including organizing, preparing, or administering medications daily, (3) a practicing community pharmacist with a license to practice in Canada and did not own a pharmacy, (4) the owner of a community pharmacy in Canada, (5) a practicing physician licensed to practice medicine in Canada, or (6) working in the insurance industry, defined as “representatives of the private (e.g., Manulife) or public insurance providers (e.g., representative of Ontario Ministry of Health and Long-Term Care)”. Patients who were not taking medications on a regular basis, caregivers who were not assisting their patients with the medication management process, and non-healthcare providers were excluded.

Once participants expressed an interest in the study, one research team member confirmed their eligibility before sending an information letter and consent form describing the purpose of the study along with an information document outlining a description of the smart products (smart multidose blister pack, NFC label, and online portal), along with a graphical representation of how real-time medication adherence data might be displayed for a test patient.

### 3.5. Data Collection

A one-on-one semi-structured virtual interview was scheduled with each participant through the Microsoft Teams platform (version: 1.5.00.27260). Interviews were conducted by one researcher (SA, SF or JI). All participants provided verbal or written consent prior to their interview. All interviews were audio/video recorded and transcribed verbatim using the NVivo transcription service (QSR International, Melbourne, Australia). The duration of the interviews ranged from 25 to 65 min. All participants were offered CAD 50 for their participation.

### 3.6. Data Analysis

The interviews were analyzed using Braun and Clark’s thematic analysis framework [30]. The interview data were organized and coded using NVivo 11 (QSR International, Melbourne, Australia). Two researchers (JI and SF) coded the first interview together. The interview was coded line by line using an inductive approach and an initial code book was developed. The researchers then coded three interviews separately. To ensure the consistency of coding, the inter-rater reliability was calculated among the two researchers (JI and SF) and was found to be 70%. During the data analysis process, constant comparison was performed and emerging codes were added to the initial codebook. Any discrepancies were resolved by discussion among the two researchers. The remaining interviews were coded independently by the two researchers (JI and SF). Codes were organized into broader categories and sub-themes and themes were developed. Since Schwartz’s value theory was used to guide interviews, codes were also mapped back to the ten values of Schwartz’s theory.

## 4. Results

### 4.1. Participants

A total of 42 stakeholders were invited to participate, of which 6 were not available to participate, the eligibility of 1 changed as they moved to a long-term care home, and 4 did not respond to the invitation to participate. Thirty-one participants from six different stakeholder groups including pharmacy owners (*n* = five), pharmacists (*n* = seven), insurance providers (*n* = five), patients (*n* = five), caregivers (*n* = five), and physicians (*n* = four) were interviewed. See Table 1 for participant demographic characteristics.

### 4.2. Themes and Sub-Themes

The interview analysis identified numerous codes as shown in Table 2. 

**Figure 2:**
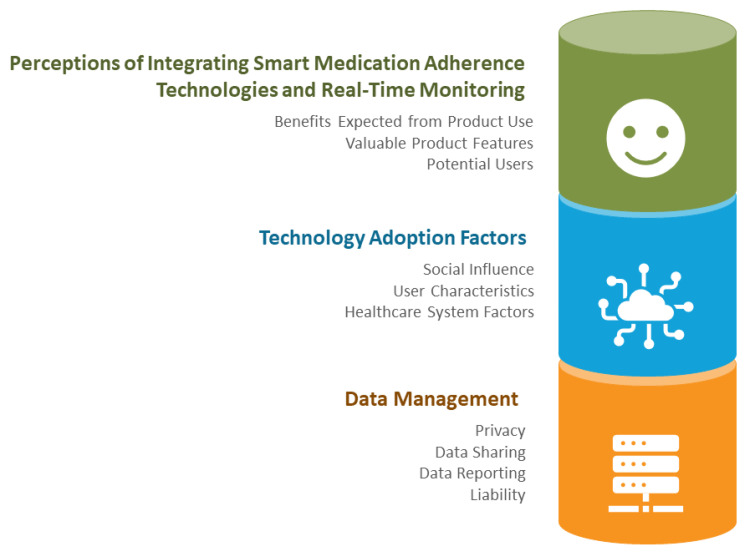
Identified Themes and Sub-Themes.

Codes were grouped into 3 themes and 10 sub-themes, as outlined in Figure 2. 

#### 4.2.1. Theme 1: Stakeholder Perceptions of Integrating Smart Medication Adherence Technologies and Real-Time Monitoring of Medication Dispensing

##### Sub-Theme 1: Benefits Expected from Product Use

All stakeholders perceived a number of benefits by incorporating smart adherence products into a patient’s daily life as well as access to real-time medication adherence monitoring. The expected benefits included improving health outcomes and fostering better communication between patients and their care providers.

“I’d say they would have less hospital visits because… they’d be taking their medication, so they’d have their chronic condition managed.”

—015 Pharmacist

“… improved like [my] relationship with my primary care provider, probably, like I feel like we would be more on the same page, I wouldn’t feel like they think that I’m this non-adherent patient who doesn’t care about my health type of thing.”

—005 Patient

Stakeholders also perceived that the use of these products might promote independent living for a patient, provide peace of mind for both patients and caregivers, and reduce caregiver stress.

“I think it would reduce the stress of the caregiver in addition to building some independence for the individual patient. I think it would be a win for all the stakeholders.”

—021 Caregiver

“If the family is involved, it gives the family some sort of a comfort knowing that they will get a message if someone in the family is not taking their medications on time so they can call them and find out what’s going on.”

—001 Pharmacy Owner

Caregivers and healthcare providers identified that the availability of real-time medication intake data could provide clinicians with useful information about their patient’s adherence.

“I think this [real-time medication intake data] would be a good thing, it’d be much easier to know whether a person is taking [medications] versus not.”

—010 Physician

Similarly, insurance providers discussed that the real-time medication intake data could be valuable in tracking the use of high-cost medications and ensuring that these medications were not being wasted.

“If you’re on a high-cost biologic drug that costs $50,000 a year, or hundreds of thousands of dollars a year. [There are only] small numbers of patients that we really need to monitor and make sure that the dollars we’re spending, like the drug is working for them.”

—029 Insurance Provider

Pharmacist stakeholders anticipated that implementing these products might improve their role in the medication management process and expand their role to becoming more clinically facing.

“Maybe they see us as really being part of the team… rather than you know, the person that just dispenses something to them in a vial, you know. We play, maybe, a little bit more of an active role in and supporting them with their journey… with their medication.”

—006 Pharmacy Owner

##### Sub-Theme 2: Valuable Product Features

All stakeholders identified numerous features that they considered would add value to smart adherence products. Stakeholders valued products that were easy and simple to use, were portable, had a locking feature, and an automated notification system to their community pharmacy for the need for refills.

“It has to be really easy to use and it should be simple.”

—009 Pharmacist

“I would like something like that… wouldn’t be too heavy to carry around.”

—027 Caregiver

“Having an option that was childproofed for those folks that have kids around or some people have grandkids or whatever around too…”

—012 Pharmacist

Pharmacists, caregivers, and patient stakeholders also discussed the value of automated inventory management at their community pharmacies through connectivity provided by medication adherence technologies.

“If you’re running low, they [pharmacy] probably know that and so they [pharmacy] can also give you your medications, right, [so] you don’t have to call them [pharmacy] all the time either.”

—004 Patient

“Uh for the pharmacist I think it would be better management for their [pharmacy] inventory and they’re like, you know, if they need to, if they’re running out of something and they have to order um from their distributors they can like, you know, plan ahead a little bit better than they can do right now. Right now, they have to rely on whatever it, of course, it’s not a live record right, like they can only calculate like you know I have dispensed until the next 30 days so if I’m running out I need to order seven days ahead of that right but if it’s live data they can like compile.”

—002 Caregiver

Stakeholders, including caregivers, pharmacists, and insurance providers also mentioned a few other valuable features in these products including large font sizes, multiple language options, and voice-controlled or screen-reading compatibility.

“The letters or the writing on it [the product] needs to be big enough so that they [users] can read it.”

—009 Pharmacist

“Irrespective of gender, age, you know, religion, you know, understanding of any kind of language.”

—023 Insurance Provider

“Read the readouts and all that stuff would certainly, I think, be very important.”

—029 Insurance Provider

##### Sub-Theme 3: Potential Users

All stakeholders identified various patient populations who could potentially benefit from these products. Among these populations were individuals experiencing memory issues or forgetfulness, older adults living alone and without caregiver support, and individuals managing complex medication regimens, or taking high-cost or critical medications.

“For those who… are in either a more complex medical or medication regime or, uh… have issues where you feel that compliance might be an issue…”

—030 Physician

“I think anyone with like mild, even mild MCI would benefit because it eliminates that that question of, you know, ‘have I taken my medication or not?’”

—021 Caregiver

Insurance providers and pharmacists particularly identified that their clients who were on short-term disability due to mental health issues could be potential users of these products and that product use could improve health outcomes and reduce their work absenteeism.

“Mental health, as an example, is an area that leads to a lot of disability costs related to mental health and mental health on its own is a rapidly increasing area of concern for employers due to again absentees and disability.”

—018 Insurance Provider

#### 4.2.2. Theme 2: Technology Adoption Factors

##### Subtheme 1: Social Influence

The adoption of medication adherence technology and real-time monitoring may be influenced by an individual’s social circle and circle of care. All four types of stakeholders postulated that a person’s pharmacist, physician or caregivers could play a significant role in integrating such technology into their medication management strategies.

“The current pharmacist that I have, it would be his… pitch that would be most important.”

—025 Patient

Similarly, a caregiver mentioned that people who were involved in the circle of care of a patient such as healthcare providers needed to take a deeper interest in order to incorporate a product into a person’s daily medication use.

“I think… the caregivers and the pharmacist and the physicians and all of that, will have to play a bigger role in this, right, as opposed to the people who are actually taking the medication themselves.”

—002 Caregiver

Stakeholders indicated that some healthcare providers in the circle of care might have more of an influence in the adoption of these technologies than others.

“If a physician is recommending it to me, like you know, like you’ve been telling me that your mom has been missing days right, so here’s the solution for you, this is what it can do and like if they can list all the things out right then I’ll be more inclined to trust that um as opposed to me going to pharmacy and then just picking one of these products and be like oh I should try this right?… So I think more influence from the physician and the pharmacist would play a better role in me buying these.”

—002 Caregiver

Similarly, pharmacists and pharmacy owners shared that it would easier to get patients to adopt a product if their caregiver were on board.

“I think if I pitched it to the caregiver and told them it was in their best interest… I think money may not be an issue if they see the value in it. If I told the patient directly, yes, there may be some resistance.”

—013 Pharmacist Owner

While the circle of care and social influence can drive the integration of such technologies for medication management by patients, it is also dependent on the willingness of patients to make this transition.

“It’s kind of like an interesting loop, because in a situation like mine, you have two people that would have to be brought on board. It would have to be the patient and the physician.”

—021 Caregiver

##### Sub-Theme 2: User Characteristics

When asked what could influence a person’s decision to incorporate a technology into their daily medication management process, stakeholders reported that there could be different aspects that influence a person’s ability to use such products including age, the user’s experience and knowledge and access to technology, learnability, and/or the user’s physical or cognitive capability to interact with the technology, which were under the sub-theme of user characteristics.

“If they have dementia and their progressing potentially, they may not be able to use this technology… and that’s where they might have to be going more into long-term care setting where someone else is administering the medication.”

—003 Pharmacist

“I think technology access would definitely be a concern for like especially for members that are older. Um for people my age, I don’t think that would really be much of a concern. I think most people have access to at least the required level of technology in order to be able to use something like this.”

—019 Patient

Stakeholders also highlighted that generational differences could impact the use of such technologies by users. For example, older adults may have difficulties in adopting such technologies as compared to younger users based on their experience with the technology from an early age. Almost all stakeholders perceived younger people to be more accustomed to technology so this group may receive the most benefit from using these products.

“Sometimes there are younger caregivers, maybe younger kids who like the technology version of many things. And we have a few persons like that. But most older adults, I think… generally stick with basics.”

—010 Physician

##### Sub-Theme 3: Healthcare System Factors

Stakeholder groups mentioned the affordability of products as one of the factors that could hinder a person’s decision to use a technology-based product. All stakeholder groups mentioned the variability among patients’ ability to afford these products.

“It depends on the situation. And I mean I have a good feel for my patients. I know…the patients that are willing to pay out of pocket and I know the patients that are not.”

—013 Pharmacy Owner

Stakeholders also discussed numerous ways to make these products affordable or have them reimbursed by public and/or private payors so patients would be more willing to consider incorporating these products for their daily medication intake.

“The machine would be nice if it was covered in some way, especially if it’s for people that meet a certain type of criteria, whether it’s age, whether there’s a lot of medication that’s hard to manage.”

—002 Caregiver

Pharmacists, pharmacy owners, and insurance providers identified some ways to reimburse these products such as using a healthcare spending account or requesting coverage under assistive device programs. Furthermore, stakeholders also discussed that there was a need to develop a pharmacy funding model for these products.

“I think moving forward, if there was a way to get some subsidized cost for this, almost like [an] assistive devices kind of program, where someone would have some cognitive concerns that there would be a little bit of something towards this technology, I think would be very useful and helpful.”

—003 Pharmacist

“I think there’s opportunity for, you know, pharmacy organizations and the pharmaceutical industry to really find the way to fund the models around adherence.”

—018 Insurance Provider

Another factor mentioned by stakeholders, including pharmacists, pharmacy owners, insurance providers, and physicians, was that more resources are required to offer these products to patients. Stakeholders mentioned that to adopt these products into their facilities, factors such as increased workload, infrastructure changes to manage pharmacy workflow, and more staff may need to be considered.

“Workflow and time are basically an issue with everything that’s new that’s implemented in pharmacy.”

—015 Pharmacist

“They’d be really worried about the increase[d] work for the amount of value that they would get out of it, just because they’re so stretched.”

—030 Physician

Insurance providers further discussed that since these are novel products, they may need to create a new product line to market these products to their plan members.

“If we were to launch this as any kind of a product that we made available, we’d have to create a product team for that. And there would be usually, there are two or three people that would be responsible for, you know, we usually create communication materials, marketing materials… We have, you know, there’s probably legal stuff that has to be signed off on. There’s compliance and regulation.”

—029 Insurance Provider

Other than resources, physicians and pharmacists mentioned that the interoperability of a product with other clinical platforms such as pharmacy dispensing software and electronic medical record platforms was also an important factor to consider for the better adoption of these products. Physicians also discussed the possibility of adopting multiple technology-based products and how developers should consider between different product interoperability.

“It would be great if moving forward there was some integration with EMR platforms. I can see that being a question from my docs and my nurse practitioners… To have something just completely link to the EMR would be fantastic so that you wouldn’t have to log on separately to another portal and that we can just have it integrated within the EMR.”

—003 Pharmacist

“If you had more than one of these types of portals, you know, like if there were different companies doing the products, which I think maybe you mentioned here… that just makes it doubly hard, right? Because then all of a sudden, you’ve got one patient with this, another patient with this. And it’s just, you know, for primary care is just so hard.”

—030 Physician

Stakeholders also inquired about real-world evidence related to the use of these products and whether these products had been tested sufficiently. Stakeholders highlighted that without real-world testing they would hesitate to adopt these novel products into practice.

“The health outcomes of these products um… and seeing what impact they actually have. To know that it is useful, and it can be useful. There has to be, you know, buy in.”

—023 Insurance Provider

Stakeholders including healthcare providers and insurance providers further discussed that if they offered these products to their patients, they wanted to see a return on their investment in the form of less drug wastage, increased revenue (for instance, by billing for medication reviews based on their review of adherence information), and a decrease in the amount of sick leave.

“I would package that as a service, because my return on investment would be through the med checks, through the follow up med checks.”

—011 Pharmacist

#### 4.2.3. Theme 3: Data Management

##### Sub-Theme 1: Privacy

Stakeholders were asked about their concerns related to the privacy and security of real-time medication adherence products and they presented mixed feelings. Some reported no concerns with privacy or security while others mentioned that they would need to be assured about the safety of how the data were being stored.

“I think that any product should be um secure… if it’s a cloud-based information transmitter or whatever, like the LibreView is… there would be information that we could share to reassure them [the patient] of the [product’s] security.”

—012 Pharmacist

“I don’t think there’s any, uh you know, impact on privacy. They already have that information. They already have your medication information, your medical history, all of that right, as long as… the app or the prod—the equipment is not collecting any other data… that it’s not supposed to collect, right, like it’s not connected to your bank.”

—002 Caregiver

##### Sub-Theme 2: Data Sharing

Stakeholders mentioned some concerns about how the data were being shared between care providers. For example, a healthcare provider commented that patient permission should be sought prior to sharing their data.

“I think anybody who has the information or has access to the information requires patient permission. They need to get that permission to have it.”

—011 Pharmacist

Additionally, caregiver stakeholders reported that if insurance companies obtained access to this information, it might impact a patient’s travel coverage as they might think ‘if I am not taking my medications as described in the product portal, I might get sick and they can deny my travel insurance’.

“Insurance companies don’t need to know everything about me, you know? Yes, I have to be truthful if I’m going to an application say for travel insurance industry to make sure I get proper coverage. But I don’t want them to run the risk of denying me coverage because I’m on something that has no bearing on my health or whatever.”

—028 Caregiver

##### Sub-Theme 3: Data Reporting

During the interview, stakeholders shared their opinions on what type of data they would like these products to report. Stakeholders mentioned that they would value the product providing actionable data-driven recommendations, rather than providing all the data in tables or figures and expecting care providers to interpret all of these data.

“I think one would have to be careful not to um unnecessarily burden physicians with information that they can’t really act on, they don’t have the time to act on versus I think this kind of detailed information may be more helpful for a caregiver or somebody who’s actually responsible for administering medicines and monitoring medicines, maybe.”

—010 Physician

Stakeholders also reiterated that they would like to see the data displayed in a graphical format, but where they could be highly customizable.

I’m just looking at this graph and if I’m looking at it and if the caregivers looking at it and they’re starting to freak out about something I can explain because we can both see.”

—011 Pharmacist

##### Sub-Theme 4: Liability

Stakeholders, particularly healthcare providers, raised many concerns about data liability. They were concerned about their ethical and legal responsibility for having access to these data, and how quickly they would be required by their professional regulations to act on them.

“I think on the surface, it sounds amazing. But I know how busy pharmacists and physicians are. Do they have opportunity to look into patient files and get alerts and do something with it?”

—018 Insurance Provider

Stakeholders were also worried about the interruption of data collection due to technology failure, false reporting by the product, and the consequences of a patient losing the device, and the impact of such events on the liability of a healthcare provider.

## 5. Discussion

### 5.1. Principal Findings

This study highlighted that all the stakeholders had limited to no knowledge and awareness about smart adherence products with the capability to track and record real-time medication intake information. This is a surprising finding, as these products have been on the market for the past two decades and one could assume that healthcare providers should be more aware of emerging adherence technology. All stakeholders recommended improving education around these products, not only for patients and caregivers, but also for healthcare providers. It is recommended that product developers focus on offering education around these products, not only for patients and caregivers but also for healthcare providers and payors.

Despite the limited knowledge, all stakeholders perceived value in using these products and the availability of real-time medication intake data. Stakeholders perceived that smart products may improve patient autonomy, reinforce patient adherence to their medication regimens due to the product’s reminder functionality, reduce caregiver burden, and improve communication and relationships within a patient’s circle of care. There are a few studies that have recently identified the benefits of products capturing real-time medication intake data. For example, a qualitative study of human immunodeficiency virus (HIV) patients and clinicians reported that real-time adherence feedback could motivate patients to adhere to their medications and improve collaboration with their healthcare providers [22]. Pharmacists and pharmacy owners valued that these products might also potentially improve or enhance their role in the medication management process as they would have more insight about a patient’s medication taking due to the accessibility of real-time medication adherence and it might help them develop personalized adherence strategies for their patients in a timely manner. Similar findings were reported by a study based in community pharmacies, which determined that pharmacists felt that having access to real-time medication intake data improved their patient interaction and helped them initiate dialogues about non-adherence based on real-time evidence [21]. Another study of older adults using a smart blister pack for medication management reported that the reminder function of the smart product made patients more aware of their medication routine and impacted their medication-taking behavior [16]. Pharmacists envisioned that the availability of real-time medication intake data might trigger a medication review, which could improve their patient–provider relationship as well as provide them with an opportunity to be reimbursed for this activity under professional services. On the other hand, pharmacy owners valued that these products might provide an opportunity to attract new clientele as they might be one of the few pharmacies offering this service at this time. A further example of how medication-taking data can impact the relationship between patients and providers is illustrated through a recent study investigating the feasibility of a tablet application among older adults [31]. This study reported that the prescription data captured by an application that notifies patients when it is time to take their medications and records the time of intake improved patient–provider communication and improved medication adherence [31].

Medication mismanagement, leading to non-adherence, is a common problem in people living with chronic diseases or mental health disorders and older adults [32,33,34,35]. Stakeholders identified a broad range of users who could benefit from the adoption of smart adherence products for their medication management process. These potential users included patients managing complex therapy regimens and those on critical or high-cost medications. Furthermore, these products may also benefit people diagnosed with chronic diseases who are managing complex therapy regimens, people living with mental health conditions or cancers, and older adults experiencing forgetfulness. A cross-sectional study investigating the prevalence and predictors of medication non-adherence among patients with asthma, hypertension, diabetes, hyperlipidemia, depression, and osteoporosis reported that 62% of patients reported forgetting to take their medications [36]. All stakeholders in our study identified community-dwelling older adults as potential users. A randomized controlled study examining the effectiveness of an electronic medication-dispensing system to improve medication adherence among older adults with chronic diseases reported that the adherence in the intervention group was significantly higher compared to the control group, but both groups had higher adherence rates than are typically seen in real-world studies, which may suggest a bias [37]. In the current study, stakeholders perceived that these products might allow patients to independently manage their medications. The inability to independently manage medications may contribute to older adults leaving their homes and moving to assisted facilities and long-term care homes. This study also identified that smart adherence products might also be an option for the caregivers of people residing in assisted facilities but not receiving medication administration assistance from the medical staff due to the high cost associated with this service. These products may have value in reducing caregiver stress by allowing caregivers to access the real-time medication intake information of their patients. This may be especially valuable for caregivers living far distances from their patients, and thus not able to support their patients physically on a regular basis. A recent observational study assessing the use of a smart medication dispenser among patients with chronic medical conditions and its impact on adherence and caregiver burden reported that caregiver burden was significantly lower after the use of a smart dispenser [38]. The stakeholders in our study also agreed that these products would benefit highly motivated patients interested in taking control of their health and younger older adults who were more familiar with the technology.

Although stakeholders perceived numerous benefits, they also emphasized that it would be essential to have more real-world evidence before adopting these products. Healthcare providers, including physicians, pharmacists, and pharmacy owners, asked for more evidence about the impact of product use on disease management and health outcomes over time, along with the overall impact on a patient’s medication management process. Similarly, insurance providers expressed their desire to have more evidence on how these products promote work productivity (for instance, reducing the total number of sick days for a patient or the duration of short-term or long-term disability leave).

Stakeholders highlighted the importance of social influence on technology adoption and emphasized that having the buy-in of all the related members of a patient’s circle of care in their decision to adopt and implement such technology is critically important. Numerous studies have reported that social influence has a significant role in technology adoption not only for patients but also for healthcare providers [39,40,41,42]. The authors recommend that healthcare providers work collaboratively with patients and their caregivers to ease the adoption of these products into a patient’s daily life.

Smart adherence products are available in many different forms and have variable features [8,9,13]. Some of the desirable features that stakeholders valued were product simplicity, portability, options to lock the product, the ability to assist with drug inventory management, the ordering of patient refills, the ability to talk to the user, multilingual options, and adjustable fonts. Other studies have identified similar results and reported that users prefer easy-to-use, simple, and portable products with the possibility of restricted access [16,20]. These findings could be useful for product developers to align their product features with users’ expectations to allow for easier adoption.

Though stakeholders found value in adopting these smart products, they also identified certain factors that could impact technology adoption. For users, these factors included affordability, cognitive and physical limitations, technology literacy, and product complexity and learnability. Stakeholders indicated that since many older adults are on a fixed income, it would be difficult for them to afford something that was not covered or subsidized. In addition, some stakeholders may not place a high value on their health-related goals and, as such, may not wish to pay out of pocket for this service. Stakeholders mentioned that in addition to the product’s base cost, there likely would be additional hidden costs, such as set-up and upgrade charges. A recent literature review reported that currently available smart medication adherence products can cost somewhere between USD 10 and USD 1500 [13]. Affordability was identified as a significant factor impacting a user’s decision to adopt a technology in this study too. For better adoption, product developers and policymakers should work closely to ensure that these products are affordable for patients.

Stakeholders also identified that the workload involved in using a product could influence the decision to adopt the technology. Research based on the usability and workload of medication adherence products by different stakeholders including older adults, caregivers, and healthcare providers has identified that there is a marked variability in the usability and workload of these products. The study reported that products such as MedReady^®^ have a higher workload as compared to other products such as TimerCap and e-pill Med Glider [17]. This information could be highly valuable for product developers to assist in designing products that are usable with less workload.

For system-wide adoption, factors such as the lack of reimbursement models, additional workload, the interoperability of the product portal with other clinical platforms and dispensing systems, and the non-clarity of the ethical and legal implications of having access to real-time medication intake data were highlighted by stakeholder groups, including healthcare providers and insurance providers. Pharmacists and pharmacy owners reported worries about the additional workload involved in offering such a service; similarly, pharmacists and physicians were concerned about the ethical and regulatory implications of this information’s availability. The amount of data collected by these products may cause challenges for healthcare providers related to data management and their ability to act on the data to support their patients’ medication taking in a timely manner. Previous research has reported similar concerns from community pharmacists [21]. These groups discussed the need to outline and manage patients’ expectations concerning their response to these data. A recent systematic review also identified data management, interoperability, and regulation issues as potential challenges to adopting healthcare technology-based products [43]. In addition, stakeholders in this study also expressed the need for a viable backup solution if the product failed or the patient lost the device. Insurance providers discussed that the lack of a reimbursement model for such a product would make it difficult for them to cover these products for their plan members

Patient and caregiver groups did not show significant concern about data privacy. However, other stakeholders stressed the importance of privacy and collecting consent for releasing and distributing data. A recent systematic review identified that data privacy and security were the main challenges that could impact the mass adoption of smart healthcare products. In our study, some stakeholders perceived that their patients might be concerned about their privacy due to their medication intake data availability [44]. Patient stakeholders stated a concern for physical privacy more than virtual data privacy. For instance, patients stated that if the size of the product was larger than or more evident than the device or system they were currently utilizing to manage their medications, they would have reservations about adopting something new. The healthcare provider group reported concerns about the sheer volume, type, and organization of the collected data. They prefer to have a customized and action-oriented adherence report rather than a detailed one. Hence, product developers should consider addressing challenges related to data privacy and security before bringing their products into the market.

To increase the value of these products to different stakeholders, future studies should focus on examining the impact of these products on medication intake behavior and the resulting impacts on health outcomes, disease management, and productivity.

### 5.2. Mapping Codes to Schwartz’s Value Theory

**Figure 3:**
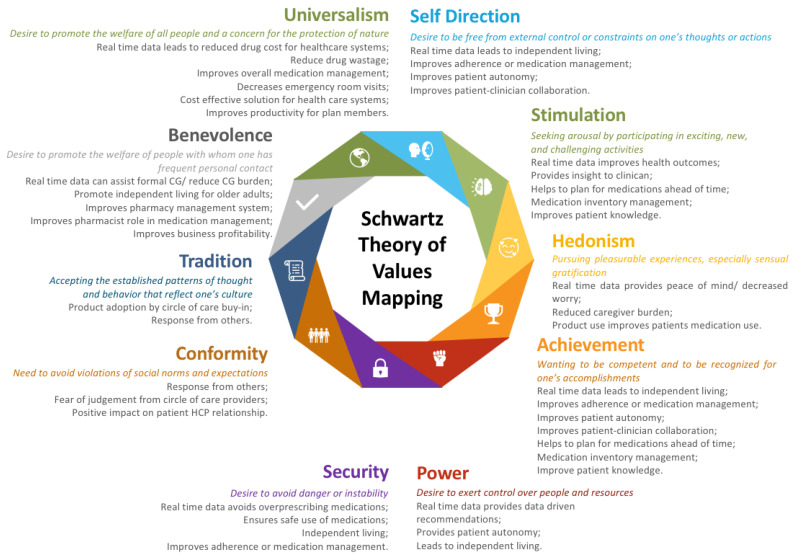
Mapping Themes and Sub-Themes to Schwartz’s Theory of Values.

In order to build a working theory from the findings of this study regarding patient preferences for medication adherence smart monitoring, we mapped the revealed codes to Schwartz’s theory of values [25]. As illustrated in Figure 3, these beliefs overlap and represent more than one specific value of Schwartz’s value theory. By mapping the interview codes to Schwartz’s theory of value, this study determined that different stakeholders place different values based on motivators or goals, which can drive product adoption. These motivators or goals should be identified and addressed for the successful adoption of a smart medication adherence product in a patient’s daily medication management process.

### 5.3. Strengths and Limitations

To the best of our knowledge, this is the first study that has explored the value different stakeholders place on smart adherence products and the availability of real-time medication intake data. The major strength of this study was the diverse sample including patients, caregivers, healthcare providers, and payors. Another highlight of this study is that we used a social sciences theoretical framework based on human values to construct the interview guide and data analysis. One limitation of our study is its small sample size. We aimed to keep the number limited to five participants and hoped to continue recruiting participants until we reached saturation. Although data saturation was reached for participant groups of pharmacists, patients, and caregivers, and no new themes emerged, we did not attain data saturation for the physicians and insurance provider groups. Unfortunately, we could not recruit more participants for these two groups within the time constraints of the study. Additionally, due to the small sample size of each sub-group, we were not able to analyze the data separately and report the findings from each group in detail, which can be considered a limitation of the study. Since most of our participants have never used or interacted with a smart adherence product, their responses are reflective of the information document provided by the research team. Another limitation of this study is the variable duration of interviews. Since some participants were only able to commit a limited amount of time for interviews, information may have been missed due to time constraints.

## 6. Conclusions

In summary, all stakeholders expressed an interest in the availability of real-time medication intake data and valued the fact that these data would allow clinicians to make timely data-driven recommendations to their patients. Stakeholders shared numerous beliefs based on motivators and goals about the expected benefits of adherence products providing real-time medication intake data access as well as identified valuable product features and factors that could impact the adoption of these technologies. These motivators and goals should be evaluated for the successful adoption of such technologies. Moreover, public and private insurance providers may need to evaluate their funding models to make these products more accessible to patients. Product developers should consider the values identified in this study for the better adoption of such products.

## Figures and Tables

**Table 1:** Sample Characteristics.

**Patient (PT) Participant Characteristics**				
**Participant ID**	**Gender**	**Age**	**Number of Medical Conditions**	**Number of Medications**
004-PT	Female	56	1	1
005-PT	Female	29	2	NA
017-PT	Female	74	2	2
025-PT	Male	78	2	2
019-PT	Male	25	2	2
**Caregiver (CG) Participant Characteristics**				
**Participant ID**	**Gender**	**Age**	**Caring for**	
002-CG	Male	32	One Parent	
021-CG	Female	60	One Parent	
022-CG	Female	51	Both Parents	
026-CG	Female	70	Both Parents	
027-CG	Female	64	Both Parents	
**Insurance Provider (IP) Participant Characteristics**				
**Participant ID**	**Work Experience**		**Educational Background**	
014-IP	10 years		Pharmacy	
018-IP	10 years		Pharmacy, Health Economics	
023-IP	N/A		Pharmacy	
028-IP	7 years		Pharmaceutical Policy	
029-IP	25 years		Pharmaceutical Consultation	
**Physician (PY) Participant Characteristics**				
**Participant ID**	**Work Experience**		**Practice**	
010-PY	>30 years		Family health team	
020-PY	37 years		Family health team	
030-PY	N/A		Academic family physician	
031-PY	13 years		Academic family physician	
**Pharmacist (P) and Pharmacy Owner (PO) Participant Characteristics**				
**Participant ID**	**Work Experience**	**Type of Practice**	**Prevalent Population**	
003-P	14 years	Chain store	Chronic pain patients, diabetic patients	
008-P	8 years	Independent	Younger adults	
009-P	5 years	Chain store	Middle-aged to senior adults	
011-P	30 years	Chain store	Senior adults	
012-P	9 years	Chain store and family health team	Senior adults	
015-P	2 years	Chain store and family health team	Mixed population	
016-P	3 years	Independent	Geriatric patients	
001-PO	6 years	Independent	Senior adults	
006-PO	5 years	Independent	Mixed population	
007-PO	6 years	Independent	Low income, senior adults	
013-PO	8 years	Independent	New families	
024-PO	6 years	Independent, multiple locations	Mixed population	

**Table 2:** Identified Themes, Sub-Themes, and Codes.

Theme	Sub-Theme	Code
Perceptions of Integrating Smart Medication Adherence Technologies and Real-Time Monitoring	Benefits Expected from Product Use	- Improved health outcomes - Improved communication - Independent living - Reinforces medication routine - Reduced burden - Peace of mind - Improves pharmacist role in medication management
	Valuable Product Features	- Simple to use - Portable - Locking feature - Drug inventory management - Talking products - Larger font - Multilingual
	Potential Users	- Long-term care homes - Patients experiencing forgetfulness - Patients on disability leave - Working people - Patients on complex, critical or high-cost medications - Highly motivated patients - Younger seniors
Technology Adoption Factors	Social Influence	- Caregiver or health care provider buy-in - Response from others
	User Characteristics	- Generational differences - Patient variation - Patients’ value of their own health - Acceptability - Limited literacy - Physical limitations - Cognitive limitations - Language barrier - Workload to use the product - Affordability - Technology limitations - Ease of use - Product familiarity - Learnability
	Healthcare System Factors	- Resources to make the product available - Marketing of the product - Product interoperability - Product testing - Real-world evidence - Reimbursement models - Hidden costs - Efficient medication access - Promotes patient productivity
Data Management	Privacy	- Concerns with privacy
	Data Sharing	- Concerns with sharing data to HCPs - Concerns for specific populations
	Liability	- Product reliability - Available back-up if product fails - Loss of device by patient - Implication for data availability
	Date Reporting	- Understanding percentage of medications taken - Actionable data-driven recommendations

## Data Availability

Not applicable.

## Supplementary Materials

The following are available online at https://www.mdpi.com/article/10.3390/pharmacy11010018/s1, Figure S1: COREQ (Consolidated Criteria for Reporting Qualitative research) Checklist, Table S1: Interview Guide based on Schwartz’s theory of values
