# Supplementary material for: Exploring the Value of Real-Time Medication Adherence Monitoring: A Qualitative Study
Source: Pharmacy (Basel). 2023 Jan 18;11(1):18. doi: 10.3390/pharmacy11010018 (PMC9967842; doi:10.3390/pharmacy11010018)
Supplement: Supplementary file 1 [file pharmacy-11-00018-s001.zip › pharmacy-2098374-supplementary.pdf]

COREQ (COnsolidated criteria for REporting Qualitative research) Checklist

A checklist of items that should be included in reports of qualitative research. You must report the page number in your manuscript where you consider each of the items listed in this checklist. If you have not included this information, either revise your manuscript accordingly before submitting or note N/A.

Topic	Item No.	Guide Questions/Description	Reported on Page No.
**Domain 1: Research team and reflexivity**			
Personal characteristics			
Interviewer/facilitator	1	Which author/s conducted the interview or focus group?	9
Credentials	2	What were the researcher's credentials? E.g. PhD, MD	NA
Occupation	3	What was their occupation at the time of the study?	NA
Gender	4	Was the researcher male or female?	Females- NR
Experience and training	5	What experience or training did the researcher have?	NA
Relationship with participants			
Relationship established	6	Was a relationship established prior to study commencement?	NA
Participant knowledge of the interviewer	7	What did the participants know about the researcher? e.g. personal goals, reasons for doing the research	NA
Interviewer characteristics	8	What characteristics were reported about the inter viewer/facilitator? e.g. Bias, assumptions, reasons and interests in the research topic	NA
**Domain 2: Study design**			
Theoretical framework			
Methodological orientation and Theory	9	What methodological orientation was stated to underpin the study? e.g. grounded theory, discourse analysis, ethnography, phenomenology, content analysis	7
Participant selection			
Sampling	10	How were participants selected? e.g. purposive, convenience, consecutive, snowball	8
Method of approach	11	How were participants approached? e.g. face-to-face, telephone, mail, email	8-9
Sample size	12	How many participants were in the study?	10
Non-participation	13	How many people refused to participate or dropped out? Reasons?	10
Setting			
Setting of data collection	14	Where was the data collected? e.g. home, clinic, workplace	9
Presence of non-participants	15	Was anyone else present besides the participants and researchers?	9
Description of sample	16	What are the important characteristics of the sample? e.g. demographic data, date	10
Data collection			
Interview guide	17	Were questions, prompts, guides provided by the authors? Was it pilot tested?	9
Repeat interviews	18	Were repeat inter views carried out? If yes, how many?	NA
Audio/visual recording	19	Did the research use audio or visual recording to collect the data?	9
Field notes	20	Were field notes made during and/or after the inter view or focus group?	NA
Duration	21	What was the duration of the inter views or focus group?	9
Data saturation	22	Was data saturation discussed?	NA
Transcripts returned	23	Were transcripts returned to participants for comment and/or	NA

Topic	Item No.	Guide Questions/Description	Reported on Page No.
		correction?	
**Domain 3: analysis and findings**			
Data analysis			
Number of data coders	24	How many data coders coded the data?	9
Description of the coding tree	25	Did authors provide a description of the coding tree?	NA
Derivation of themes	26	Were themes identified in advance or derived from the data?	9
Software	27	What software, if applicable, was used to manage the data?	9
Participant checking	28	Did participants provide feedback on the findings?	NA
Reporting			
Quotations presented	29	Were participant quotations presented to illustrate the themes/findings? Was each quotation identified? e.g. participant number	11-23
Data and findings consistent	30	Was there consistency between the data presented and the findings?	NA
Clarity of major themes	31	Were major themes clearly presented in the findings?	NA
Clarity of minor themes	32	Is there a description of diverse cases or discussion of minor themes?	NA

Figure S1 COREQ (Consolidated Criteria for Reporting Qualitative research) Checklist.

Table S1. Interview Guide based on Schwartz's theory of values [25].

Background information	Can you tell us a little about yourself?	
Views and knowledge	What have you heard about products that can record and track real-time medication intake?<br>Tell us about the products that you have heard of<br>What do you think about these products? And why do you think that?	
Values	**Definition**	**Question**
Self-direction	Desire to be free from external control or constraints on one's thoughts or actions.	Do you see yourself or your patients using these products?<br>When would you discourage/encourage the use of these products for yourself/your loved one/your patient/your insuree?<br>What type of patient population would be suitable or not suitable for these products? Why or why not?<br>How do you see these products fitting into your/your patient's/your insuree's daily schedule?
Stimulation	Seeking arousal by participating in exciting, new, and challenging activities	How would these products change the way you manage your or your patient's medication intake?<br>How do you think the feedback about such products will impact your ability to recommend/offer/cover these products to your patients and their families?

Hedonism	Pursuing pleasurable experiences, especially sensual gratification	What are the features that you/your patient/your loved one/your insuree would like to see in such products or will make you want to use the product?<br>How would you feel about working more closely with your/your loved one's HCP about managing your/your loved one's medication intake?<br>How would you feel about collaborating with other HCPs/CG/patients via the use of these products?
Achievement	Wanting to be competent and to be recognized for one's accomplishments	How do you think these products will help you/your loved one/your patient to manage medications?<br>How do you think these products will ultimately help or hinder whether you/your loved one/patient/etc., manage your/their health?<br>How do you think it will affect quality of life?
Power	Desire to exert control over people and resources	How do you think the use of these products will impact your/your loved one's/your patient/insuree's ability to be independent/autonomous of their medication intake?<br>How do you think the availability of medication intake information and adherence impact your conversations with your physician/pharmacist/patient/insuree?<br>What kind of return on investment/benefit do you think is possible if you bought/prescribed/insured this product for yourself/patient/loved one/insuree?
Security	Desire to avoid danger or instability	How do you think the use of these products will impact your/your patient's safety related to medication intake? OR put them in danger?<br>What do you think about the impact on privacy of with these products?
Conformity	Need to avoid violations of social norms and expectations	How would others around you feel/think about you using/recommending these products?<br>What do you think your friends/family/colleagues/industry would say or think if they saw you use/prescribe/insure such products?
Tradition	Accepting the established patterns of thought and behavior that reflect one's culture	How would others around you/your patient/insuree feel/think about you using/recommending/insuring these products over standard interventions such as the traditional blister pack, Dossett box, etc.

Benevolence	Desire to promote the welfare of people with whom one has frequent personal contact	What kind of usefulness if any, do you see with these products for you or your patients'/insuree medication management process?<br>How would these products impact your ability to care for your patient/loved one?<br>What kind of issues do you anticipate with these products?
Universalism	Desire to promote the welfare of all people (including strangers) and a concern for the protection of nature	What would drive/encourage you to use/purchase/recommend/provide coverage for this technology to your own use/your patients/your clients?<br>How do you think the use of these products will help collaboration with other professionals?
